# Orally administered β-glucan improves the hemolytic activity of the complement system in horses

**DOI:** 10.14202/vetworld.2021.835-840

**Published:** 2021-04-05

**Authors:** Taline Scalco Picetti, Lucas de Figueiredo Soveral, Rovian Miotto, Luana Marina Scheer Erpen, Yasmin Kreutz, João Antônio Guizzo, Rafael Frandoloso, Luiz Carlos Kreutz

**Affiliations:** 1 Laboratório de Microbiologia e Imunologia Avançada, Faculdade de Agronomia e Medicina Veterinária, Universidade de Passo Fundo, 99052-900 Passo Fundo, RS, Brazil; 2 Programa de Pós-Graduação em Medicina Veterinária Preventiva, Universidade Federal de Santa Maria, 97105-900 Santa Maria, RS, Brazil

**Keywords:** animals, glucans, monocytes, muramidase

## Abstract

**Background and Aim:**

Immune-modulating molecules mainly act on innate immune cells, which are central to early defense against invading pathogens and contribute to developing adaptive immunity. Yeast-extracted β-glucan, a model immune-modulating molecule, is widely used in several animal species; however, its effect on horse immune parameters has not been thoroughly investigated yet. This study aimed to evaluate the effects of orally administered β-glucan on selected innate immune parameters in horses.

**Materials and Methods:**

Eighteen thoroughbred horses were assigned equally into three groups as follows: One control group (no β-glucan) and two β-glucan experimental groups (one received 125 mg and the other 2 g of β-glucan per day for 28 days). Blood samples were collected before and at the end of the experiment for hematological analysis, whole blood phagocytosis, respiratory burst assays, and to assess the serum lysozyme and complement hemolytic activities.

**Results:**

At the end of the experiment, significant decreases (p<0.05) in monocyte numbers were observed in the control horses (258.8±45.9 vs. 115.3±41.5) and in those fed 125 mg/day of β-glucan (208.8±72.3 vs. 99.2±60.7), whereas a significant increase in numbers was noted in the horses that were fed 2 g/day of β-glucan (303.5±45.8 vs. 429.8±86.0; p<0.05). The natural hemolytic activity of the complement was higher only in horses fed 2 g/day of β-glucan (p=0.018) compared to the other groups. The hemolytic activity in the classical pathway was higher in those fed 125 mg/day (p=0.0035) and 2 g/day of β-glucan (p=0.0001).

**Conclusion:**

β-glucan improves important innate immune parameters and might be fed to horses before stressful events.

## Introduction

Stressful situations such as parturition, weaning, training exercises, frequent transportation, and competition events have short-term negative effects on the immune response [1,2] and might contribute to disease outbreaks in horses. The possibility of improving innate and acquired immune responses is central to good health and performance, and the use of immune modulators in horses is a subject of great interest to researchers, practitioners, and horse owners [3-6].

The use of immune-modulating molecules in horses has been poorly investigated and limited to few active components. Most widely used immune modulators contain molecules derived from pathogens, known as pathogen-associated molecular pattern (PAMP) molecules, which interact with pathogen recognition receptors (PRRs) found on the membrane surface or intracellular compartments of immune cells [7]. The interaction between PAMPs and PRRs triggers the activation of immune cells leading to activities such as improved phagocytosis and respiratory burst and the expression of key cytokines that coordinate subsequent immune events. In horses, the administration of *Parapoxvirus ovis* (PPVO) stimulates the mRNA expression levels of interferon (*IFN*)*α*, *IFNβ*, and *IFNγ* in peripheral blood mononuclear cells [5,8-10] and improves the oxidative burst and phagocytosis of *Rhodococcus equi* by neutrophils [9]. In contrast, PPVO does not affect the development of cell-mediated immunity in weaned foals [11] or the incidence of pneumonia [5] in farms endemic for *R*. *equi*. The effects of *Propionibacterium acnes* (PA) on horse neutrophil respiratory burst and phagocytosis [5] have been investigated, but are not clear yet. Horses inoculated with *Streptococcus zooepidemicus* demonstrated a reduced incidence of endometritis and a downregulation in the expression levels of IL-1b on the endometrial cells after treatment with mycobacterial cell wall extract (MCWE) [4]. PPVO, PA, and MCWE were parenterally administered in these studies, which limit their daily application over longer durations.

Ideally, immune-modulating molecules should be orally administered with food. The yeast-extracted β-glucan has been widely used as a food additive to improve the innate immune system of several animal species [12,13]. β-glucan has been shown to improve the overall resistance by increasing respiratory burst, phagocytosis, lysozyme, and complement activity in some animal species [14]. However, its effect on horses has been poorly investigated.

In this study, we aimed to investigate the effects of β-glucan, as a food additive, on blood leukocytes and selected innate immune parameters in English thoroughbred horses under regular training conditions.

## Materials and Methods

### Ethical approval

The study was carried out in accordance with the regulations of the National Council for the Control of Animal Experimentation and approved by the Committee of Ethics in Animal Experimentation (CEUA protocol number 016/2018) of the Universidade de Passo Fundo.

### Study period and location

The experiment was carried out in March 2019 using horses that were housed and trained in a local horse farm at the municipality of Passo Fundo, Rio Grande do Sul (28° 15′ 40″ S, 52° 24′ 30″ W and 680 m altitude). Blood samples were analyzed at the Laboratório de Microbiologia e Imunologia Avançada, Universidade de Passo Fundo.

### Animals and experimental design

Eighteen 2-year-old English thoroughbred horses (7 females and 11 males) were used in this study. All horses were considered healthy, according to physical and hematological examinations, and were treated for intestinal parasites followed by vaccinations according to the farm protocol. The animals were placed in individual stalls and on an exercise routine for 3 months when β-glucan (Biorigin, Brazil) was added to their diet. The diet was composed of a balanced commercial ration, Tifton-85 hay, alfalfa, and water *ad libitum*. The horses were randomly and equally assigned to three groups: A control group (no β-glucan) and two experimental groups which received 125 mg/day and 2 g/day of β-glucan, respectively, in the food for 28 days. All animals were kept in individual stalls and underwent an exercise regime once a day using the same protocol throughout the experimental period.

### Blood sampling and hematological analysis

Peripheral blood samples were collected through jugular venipuncture before the start of feeding β-glucan (day 0) and at the end of the experiment (day 28). Blood samples for hematological analysis were aliquoted into EDTA tubes; samples used for whole blood phagocytosis and respiratory burst assays were aliquoted into heparin tubes and immediately placed on ice. A third blood aliquot was allowed to coagulate at 23ºC to 27ºC for 2 h to obtain serum for the immunological assays. After coagulation, the serum was carefully removed and frozen (−20°C) until further analysis. The hematological parameters were immediately evaluated on whole blood samples, using the PocH-100iV equipment (Sysmex, Brazil), and on blood smears stained with Wright-Giemsa.

### Whole blood phagocytic and respiratory burst assays

Bacterial phagocytosis and respiratory burst assays were performed using heparinized whole blood samples. The presence of anti-bacteria antibodies in horse serum could affect the outcome of the whole blood phagocytic assay in different animals; therefore, a dot blot assay was used to determine the presence of antibodies to selected bacteria (*Escherichia coli*, *Staphylococcus aureus*, *Streptococcus equi*, and *Glaesserella parasuis*) in the horses. Lack of serum reactivity on the dot blot assay was observed only with *E*. *coli*. Consequently, fluorescein isothiocyanate (FITC; Sigma-Aldrich, Brazil)-labeled *E*. *coli* (ATCC 25922) was used in the phagocytic and respiratory burst assays. In brief, *E*. *coli* was grown on LB media at 37°C under constant agitation (200 rpm) until the optical density (OD) of the growing media reached 0.7 at 600 nm. Then, the bacteria were inactivated by adding formalin to a final concentration of 0.5% and kept under agitation at 37°C for an additional 12 h. The inactivated bacteria were harvested through centrifugation (20 min, 4500 rpm), washed 3 times with phosphate-buffered saline (PBS, pH 7,4), and counted using flow cytometry. FITC labeling was performed using 1 mg of FITC per 1×10^9^ bacteria in the dark (30 min at 22°C), under constant agitation (300 rpm). The bacteria were then washed again to remove excess FITC, diluted with PBS (containing 1% of albumin), and stored in the dark at 4°C. The phagocytic assay was carried out as described previously [15], with slight modifications. In brief, 200 mL of whole heparinized blood was mixed with 2×10^7^ FITC-labeled *E*. *coli* (25 bacteria per leucocyte) and incubated at 37°C for 10 min. Then, the samples were placed on ice and mixed with red blood cell lysis buffer (Sigma-Aldrich) for 10 min. The samples were analyzed using BD FacsVerse flow cytometry (BD Life Sciences, Brazil) with forward scatter (FSC)-side scatter (SSC) linear amplification and biexponential fluorescence. Leukocytes were analyzed according to the size and complexity (FSC×SSC); the phagocytic cells (polymorphonuclear and monocytes) were identified within the blood leukocyte population, and the fluorescence intensity and percentile of phagocytic cells (FL1 channel) were evaluated.

The respiratory burst assay was carried out similar to the whole blood phagocytic assay, but using non-labeled *E*. *coli* [15]. Non-labeled *E*. *coli* and 10 mL of 2′,7′-dichlorofluorescein diacetate (DCFH-DA, 500 mM, Sigma-Aldrich) were added simultaneously to whole blood aliquots (200 mL), and the mixture was incubated for 10 min at 37°C. The samples were ice refrigerated, and erythrocytes were lysed before evaluation using flow cytometry as described earlier.

### Serum lysozyme activity determination

Lysozyme activity in horse serum was determined through the turbidimetric assay using *Micrococcus lysodeikticus*, as described previously [16]. Briefly, 20 mL of horse serum diluted in PBS (1:20) was mixed with 180 mL of *M*. *lysodeikticus* (OD 0.5 at 450 nm) in flat-bottomed plates. The samples were incubated at 23°C, and OD was measured again at 1 and 4 min using an enzyme-linked immunosorbent assay plate reader (SynergyÔ H1, BioTek, USA). The standard curve of lysozyme white chicken egg (Sigma-Aldrich, USA) was used as positive control, and lysozyme activity was calculated as follows: ([DOD (4-1 min)/3]/0.001)×100, where a reduction of 0.001 on the OD reading corresponded to 1 IU/mL.

### Serum complement activity

The hemolytic activity of the alternative and classical complement pathways in horse serum was evaluated. For the alternative pathway, 100 mL of horse serum was diluted (factor 2) in HEPES-buffered saline (HBS, 150 mM NaCl, 135 nM CaCl_2_, 1 mM MgCl_2_, 10 mM HEPES, pH 7.2) containing 0.01% gelatin and 1 mM ethylene-glycol-bis-tetraacetic acid (EGTA) [17] using “U”-shaped 96-well plates. The calcium-chelating EGTA was added to prevent the activation of the calcium-dependent classical complement pathway. Then, 50 mL of chicken erythrocytes (1%) were added and the plates incubated at 37°C for 1 h with gentle shaking every 10 min. The plate was centrifuged (230×*g*) for 5 min at 4°C, and the supernatant was transferred to a flat-bottomed plate for OD reading at 412 nm (SynergyÔ H1, BioTek, USA). Erythrocytes incubated with sterile water, or HBS only, presented 100% and 0% of hemolysis, respectively, which was used to calculate the hemolytic activity in each sample.

The hemolytic activity of the classical pathway was evaluated using a hemolytic solution (HS) containing chicken erythrocytes opsonized with rabbit anti-chicken antibodies. In brief, a rabbit was immunized through intravenous injection of 10% chicken erythrocyte solution (1 mL/kg of body weight) on days 1, 2, 3, 4, 7, and 11. Blood was collected through intracardiac puncture, under anesthesia, on day 14. The HS was prepared by mixing chicken erythrocytes (2%) solution with an equal volume of HBS containing rabbit anti-chicken antibody (diluted to 1:1.500), incubated for 10 min at 37°C, and then stored on ice until further use. For the classical complement hemolytic assay [17], 100 mL of horse serum was diluted (factor 2) in HBS containing 0.01% gelatin (no EGTA) using “U”-shaped 96-well plates, mixed with equal amounts of HS, and incubated at 37°C for 1 h with gentle mixing every 10 min. The plate was centrifuged, and the supernatant was transferred to flat-bottomed 96-well plates and read at 412 nm. Erythrocytes incubated with sterile water, or HBS only, presented 100% and 0% of hemolysis, respectively, and were used to calculate the hemolytic activity in allsamples.The OD values obtained by reading serum samples prior to the CF test were discounted from the final reading.

### Statistical analysis

The data were evaluated using Shapiro–Wilk test and found to have a normal distribution. Blood parameters were analyzed using two-way analysis of variance (ANOVA) followed by Tukey’s post-test; complement data were evaluated using two-way ANOVA followed by Dunnett’s multiple comparison test. Data were plotted using the GraphPad Prism Software v7 (GraphPad Software, Inc., San Diego, CA, USA). The results are expressed as the mean ± standard error of the mean, and p≤0.05 was considered statistically significant.

## Results

### Effect of β-glucan on blood cells

The hematological parameters were evaluated before (day 0) and 28 days after feeding the horses with β-glucan (125 mg/day or 2 g/day). The number of monocytes was significantly higher (p<0.05) on day 28 in horses that were fed 2 g/day of β-glucan (Table-1); alternatively, on those horses, total serum protein levels were lower on day 28 compared with day 0.

**Table 1:** Hematological parameters of horses fed β-glucan. Blood samples were collected before (day 0) or after the feeding regimen (day 28). The data represent the mean±SEM (n=6).

Blood parameter	Control		β-glucan			

			125 mg/day		2 g/day	
		
	Day 0	Day 28	Day 0	Day 28	Day 0	Day 28
Erythrocytes (10^6^/mL)	10.9±0.45	10.5±0.54	11.2±0.36	10.5±0.52	10.9±0.49	10.2±0.31
Hemoglobin (g/l)	14.1±0.36	13.6±0.64	13.6±0.27	12.8±0.39	13.4±0.41	12.5±0.21
Hematocrit (%)	42.6±1.17	41.3±1.95	42.4±0.85	39.8±1.24	41.3±1.38	38.9±0.68
VHS (mm/h)	39.2±1.16	39.5±1.16	37.9±0.85	37.9±0.81	33.9±4.03	39.2±0.82
HCM (pg)	13.0±0.50	13±0.55	12.4±0.36	12.2±0.30	12.4±0.28	12.4±0.28
CHCM (g/dl)	33.1±0.35	32.9±0.40	32.1±0.13	32.1±0.14	32.3±0.24	32.3±0.31
Total leukocytes (μL)	10.1±1.10	8.4±487	9.167±300	8.000±619	8.1±319	7.1±305
Bastonated neutrophils (μL)	48.3±21.62	13.2±12.01	28.3±25.86	0±0	13.6±12.47	11.7±10.65
Segmented neutrophils (μL)	6296±1227.0	3876.6±405.3	4593.7±264.4	3325.2±326.3	3994.2±317.5	2921.8±130.8
Eosinophils (μL)	203.8±95.36	152.2±40.32	133.3±55.55	149±53.11	86.7±40.35	104.8±12.58
Basophils (μL)	35.3±0	0±0	0±20.94	0±0	0±0	0±0
Lymphocytes (μL)	6107.6±354.8	4186.8±526.5	4202.5±2400	4426.6±253.8	3718.7±336.8	3665±304.12
Monocytes (μL)	258.8±45.9	115.3±41.5^A^	208.8±72.3	99.2±60.7^A^	303.5±45.8	429.8±86.0^B^
Platelets count (μL)	182.0±26.4	173.7±11.3	255.3±14.7	219.333±14.0	189.000±8.9	211.33±9.9
Total protein (g/dl)	8.1±0.09^a^	7.6±0.13^b^	7.9±0.09	7.6±0.09	8±0.08^a^	7.5±0.08^b^

Significant differences (p<0.05) within the same group are represented by small letters and differences between groups are represented by capital letters. HCM=Mean Corpuscular Hemoglobin, VHS=, Erythrocyte Sedimentation Rate CHCM=Mean Corpuscular Hemoglobin Concentration

### Whole blood phagocytosis and respiratory burst assays

Phagocytosis was measured as the percentile of phagocytic cells associated with FITC-labeled bacteria. The percentile of cells with attached bacteria was similar among groups on days 0 and 28 and was not altered by β-glucan supplementation (data not shown). Similarly, the percentile of fluorescent cells evaluated in the respiratory burst assay was similar on days 0 and 28 in all groups and was not affected by β-glucan feeding (data not shown).

### Serum lysozyme and both alternative and classical complement hemolytic activities

**Figure-1:**
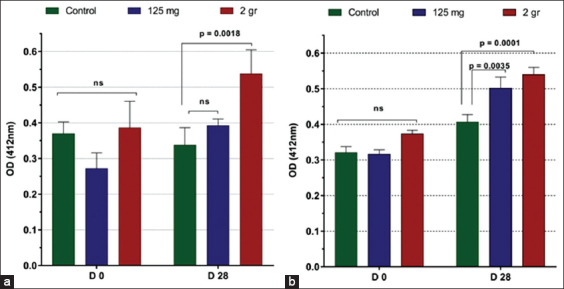
Horse serum (a) alternative and (b) classical complement pathway activation on chicken erythrocytes. Data are expressed as the optical density (OD_412 nm_) mean±SEM (n=6) obtained from the supernatant of lysed erythrocytes. Supernatant from 100% hemolysis had an OD_412_ of 0.750. Significant differences among treatment are indicated (p-value).

Serum lysozyme activity was measured on days 0 and 28; no alterations following β-glucan treatment were detected (not shown). In contrast, the hemolytic activity of the alternative complement was significantly higher on day 28 in horses that received 2 g of β-glucan (Figure-1a). Furthermore, the hemolytic activity in the classical complement pathway was higher on day 28 in the two experimental groups of horses (Figure-1b).

## Discussion

β-glucan is a linear polysaccharide extracted from the cell walls of yeast, algae, and fungi [12] that can function as PAMPs and be administered as food additives to animals to improve both innate and acquired immunity [14]. The interaction between β-glucan and its cognate receptor on immune cells (e.g., macrophage and dendritic cells) triggers the expression of cytokines, which orchestrates innate and acquired immune responses [13]. The immune-modulating effects of β-glucan have been demonstrated in several animal species [13], including fish [18]; however, their effect in horses has not been demonstrated so far. The advantage of β-glucan over other PAMPs evaluated in horses is that it can be administered orally, rather than through parenteral injections. However, the effects of β-glucans are better noticed after a prolonged period of feeding; moreover, the *in vivo* effects are rather discrete compared with the *in vitro* effects [19]. Nonetheless, this study provides data favoring the use of β-glucan as an innate immune modulator in horses.

β-glucan is an insoluble non-digestive carbohydrate [14], which interacts with pinocytic microfold M cells located in the small intestine[12] and immune cells, such as macrophages in Peyer’s patches. Macrophage-processed β-glucan can be transported to the lymph nodes, spleen, and bone marrow [20], enhancing the expression of several immune-related genes, including the complement pathway-related genes [21]. The feeding of algae-derived β-glucan to weaned *E*. *coli* challenged piglets resulted in a reduction in the transcellular permeability of the gut [22], delay in the onset of diarrhea, and a reduction in the number of blood neutrophils. In the current study, β-glucan had no significant effect on the number of blood leukocytes, except for monocytes, in the horses. The monocyte count on day 28 was lower than that on day 0 in horses that were fed 125 mg/day of β-glucan. However, a reduction in monocyte counts was observed in the control group as well; hence, it cannot be attributed to β-glucan. In contrast, horses fed with 2 g of β-glucan had a significantly higher number of blood monocytes at the end of the feeding regimen. Blood monocytes are central to innate immunity; they migrate from blood to tissues, monitor the invading pathogens, and secrete cytokines that direct the appropriate immune responses [23].

In addition, monocytes secrete anti-microbial peptides, lysozymes, and complement components that further improve the innate defense [24]. In the present study, serum lysozyme activity was highly variable within and among the groups, but not significantly different or affected by β-glucan treatment. However, the hemolytic activity of the alternative and classical complement system was higher in horses that received β-glucan. Complement components are produced mostly by hepatocytes and mononuclear cells. In mammals, the complement system is central to innate and acquired immunity. On activation, key components of the complement system are activated, and a cascade of enzymatic reactions is initiated that results in the formation of the membrane attack complex on the surface of the target pathogen [25]. In addition, complement activation leads to the production of several soluble intermediary components with important roles in immune cell chemotaxis, microbial opsonization, destruction, and phagocytosis [26], mostly by neutrophils that are attracted to the site of infection. The complement system in horses and its role in protecting against invading pathogens have been poorly investigated. Early studies indicated that horses had a potent alternative complement pathway capable of readily lysing rabbit erythrocytes [27] but not sheep erythrocytes [28]. Thus, the efficacy of the complement system in horses might depend on the pathway activated and the target antigen. In the current study, we investigated the activation of both the alternative and classical complement pathways and found that feeding horses with β-glucan had a significant effect on the hemolytic activity against chicken erythrocytes (target cells) during both alternative and classical pathway activations. Complement activation through the alternative pathway begins with the spontaneous deposition of a key complement component, the C3b molecule, on the target cell surface, whereas classical pathway activation occurs after the binding of the C1q molecule to antibodies attached to the target cell [25]. Thus, the complement system plays a major role in innate and acquired immunity in most animal species. Data on the effect of β-glucan on the hemolytic activity of the complement system in mammals are scarce, probably due to challenges in standardizing the hemolytic assay to each species. Alternatively, non-hemolytic assays that measure complement activation by detecting the byproduct depend on specific reagents and anti-complement component antibodies that are not readily available.

## Conclusion

The use of immune modulators in horses is in its incipient stage and needs to be explored further. Some immune modulation therapies and compounds have proven beneficial, but many others lack scientific verification. In this study, we demonstrated that feeding horses with β-glucan improve immune-related cells and booster complement activation, a major component of humoral immunity in mammals. These findings warrant further investigations, particularly concerning the dosage and treatment regimen. Additional tools are required to investigate the effects of β-glucan on the expression of the immune-related genes *in vivo* and *in vitro* to better understand its potential use as an immune modulator in horses.

## Authors’ Contributions

TSP and LCK: Conception and design of the study. TSP, LFS, JAG, RM, LMSE, and YK: Acquisition of the data. LCK, TSP, JAG, and RF: Analysis and interpretation of the data. TCP, RF, and LCK: Analyzed the data and drafted the manuscript. LCK: Critical revision of the manuscript. All authors read and approved the final manuscript.
